# Supplementary material for: A novel methodological approach to SaaS churn prediction using whale optimization algorithm
Source: PLoS One. 2025 May 13;20(5):e0319998. doi: 10.1371/journal.pone.0319998 (PMC12074543; doi:10.1371/journal.pone.0319998)
Supplement: Attachment — Submitted filename: Authors Responses to Reviews.docx [file pone.0319998.s001.docx]

Dear Editor,

Thank you very much for your valuable time organizing our paper's review. We are very grateful to the esteemed reviewers for their thorough assessments and thoughtful suggestions on our manuscript. The comments have been fair, encouraging, constructive, and valuable. We have carefully considered all the reviewers' comments in our revised version. Please find our revision summary and responses to all reviewers below. In this response and the revised paper, we have clarified our ideas, highlighted the motivation, reorganized the paper, and addressed the reviewers' concerns and misunderstandings. We have responded to all the reviewers' comments. Thank you again for kindly reviewing our work.

We are uploading our responses to the comments and an updated manuscript, with changes highlighted in yellow.

Best regards,

**Reviewer#1, Concern # 1:**

Your manuscript titled "A Novel Methodological Approach to SaaS Churn Prediction Using Whale Optimization Algorithm" presents a technically sound and innovative approach to customer churn prediction in the SaaS sector. The use of the Whale Optimization Algorithm (WOA) for feature selection and the comprehensive evaluation of multiple predictive models contribute significantly to the academic discourse on churn analysis. The study is well-structured, and the methodology is robust, making your findings valuable for both academic and practical applications.

**Author Response:** *Thank you for your positive feedback. We appreciate your recognition of our approach and methodology.*

**Reviewer#1, Concern # 2:**

Technical Soundness and Data Support: Your research is methodologically sound, with rigorous experimental design and execution. The comparative analysis of different datasets (WOA-reduced, full-variable, and chi-squared-derived) is thorough, and the use of multiple performance metrics provides a comprehensive evaluation of model effectiveness. The conclusions drawn are well-supported by the data, and the identification of significant features for churn prediction is both novel and practical.

**Author Response:** *We greatly appreciate your positive remarks on our methodology and feature analysis. Your recognition of the study’s rigor is encouraging.*

**Reviewer#1, Concern # 3:**

2. Statistical Analysis: The statistical analysis in your manuscript is appropriately conducted. The use of 10-fold cross-validation adds rigor to your model evaluations, and the performance metrics employed are standard and relevant for this type of analysis. However, the inclusion of statistical significance tests (e.g., t-tests or ANOVA) to compare the performance of different models could further strengthen your analysis. While the current presentation of results is clear, adding these tests would provide additional confidence in the observed differences between models.

**Author Response:***. Thank you for your valuable feedback on the statistical analysis. In response to your suggestion regarding the inclusion of statistical significance tests to further strengthen the analysis, we have taken this into consideration and have made improvements to the manuscript.*

*Specifically, we have incorporated the* ***Friedman, non-parametric statistical test*** *to evaluate whether there are significant differences in performance across the models. The Friedman test is well-suited for this analysis, as it is designed for comparing multiple models across datasets [96],[97]. Following this, we performed the* ***Nemenyi post-hoc test*** *to identify which specific pairs of models exhibited significant differences. The results of this test are presented in the manuscript via a heatmap, where the degree of significance is visually represented.*

*As suggested, these additions provide further statistical rigor to the analysis and offer additional confidence in the observed differences between models. Thank you again for your insightful suggestion, which has led to an enhanced presentation of the study.*

*In manuscript:*

For further statistical validation, we employed the Friedman test to determine if there is significant differences in performance among the models. The Friedman test is one of the best non-parametric statistical tests to detect differences between multiple models across multiple datasets [96,97]. The test revealed below the significant level in the models' AUC performances (p= 0.001190), prompting us to conduct the Nemenyi post-hoc test to pinpoint the specific model pairs with significant differences. The results of the Nemenyi test were presented in a heatmap, with each cell displaying the p-value for a pairwise comparison between the two models. The color scale ranging from blue to red reflects the degree of significance, where blue cells denote lower p-values that highlight significant differences between models, and red cells indicate non-significant differences.

*Figure 5. Friedman and Nemenyi test for comparative analysis of different models*

**Reviewer#1, Concern # 4:**

3. Data Availability: Your manuscript mentions that the data underlying the findings cannot be shared publicly due to privacy concerns but can be accessed by qualified researchers upon request. While this is understandable, it does not fully align with PLOS ONE’s data availability policy, which emphasizes the need for data to be fully available without restriction. To better comply with this policy, you might consider anonymizing the data or providing more detailed instructions on how researchers can request access to the data.

**Author Response:** *Thank you for your feedback. We have discussed the data-sharing concerns with the company, and they have confirmed that, due to privacy regulations and company policies, the data cannot be shared publicly. However, we understand the importance of data availability and are committed to transparency within the bounds of these constraints.*

*Researchers interested in accessing the data may contact the corresponding author, who will facilitate communication regarding data access requests with the company. We appreciate your understanding and support in balancing privacy concerns with the goals of university-industry collaboration.*

**Reviewer#1, Concern # 5:**

4. Presentation and Language: The manuscript is generally well-written and presented in standard English, making it accessible to a broad audience. However, there are a few minor grammatical and typographical errors that should be addressed during revision. Additionally, some sentences could be restructured to improve clarity and readability. Consistency in terminology, particularly regarding feature selection and dataset descriptions, would also enhance the manuscript's overall coherence.

**Author Response**: *Thank you for your detailed feedback on the manuscript’s presentation and language. We addressed minor grammatical and typographical errors and restructured sentences to enhance clarity and readability. We also ensured consistency in terminology related to feature selection and dataset descriptions to improve the manuscript's coherence.*

**Reviewer#1, Concern # 6:**

Clarity in Methodological Explanation: While the methodological approach is generally clear, providing more detailed explanations of certain steps, such as the specific criteria used for feature selection with WOA, would further clarify your approach.

**Author Response:** *Thank you for your constructive feedback. To enhance the clarity of our methodological approach, we included a more detailed explanation of the criteria used for feature selection with the Whale Optimization Algorithm (WOA).*

*In manuscript:*

In WOA for feature selection, each feature subset consisting of features from the original set is represented as a position of a whale. The algorithm begins with a population of randomly generated feature subsets, and each solution is evaluated using the fitness function. The ideal solution is one that selects fewer features while maintaining higher classification accuracy [65]. WOA iterates through a series of steps in which the solutions update their positions, simulating the bubble-net hunting behavior and searching for prey. During each iteration, solutions adjust their positions based on either the shrinking encircling mechanism or the spiral model [65]. The shrinking encircling mechanism aims to balance exploration and exploitation using random vectors. The process continues until the stopping criteria is met.

**Reviewer#1, Concern # 7:**

Ethical Considerations: There are no apparent ethical concerns regarding dual publication or research ethics based on the provided information. Your acknowledgment of privacy concerns related to data sharing is appropriate, though further clarification on how others can access the data is recommended.

**Author Response:** *Thank you for your assessment. We appreciate your acknowledgment of our privacy considerations. We further clarified the data access procedures to ensure transparency and compliance with ethical standards.*

**Reviewer#1, Concern # 8:**

Conclusion: Your manuscript makes a valuable contribution to the literature on SaaS churn prediction and feature selection methods. By addressing the minor language issues and considering the suggestions for data availability and statistical analysis, your manuscript would be well-positioned for publication. I encourage you to make these revisions to enhance the clarity, rigor, and compliance with journal policies.

Thank you for your contribution to the field.

**Author Response:** *Thank you for your kind remarks and constructive feedback. We proofread the minor language issues. About the data availability, we discussed your suggestion in the “Reviewer 1 Concern 4” part; due to privacy regulations and company policies, the data cannot be shared publicly. We incorporated the recommended statistical analyses to strengthen the manuscript. We appreciate your guidance and support.*

**Reviewer#2, Concern # 1:**

The authors developed a method to SaaS Churn Prediction Using meta-heuristic: Whale Optimization Algorithm to identify critical variables rather than all variables affecting churn rates. The topic is interesting and the literature review is sufficient. The results are clearly presented. The paper could be considered for publication after minor revisions.

**Author Response:** *Thank you for your positive feedback on our manuscript and for recognizing the interest and clarity of our study. We addressed the minor revisions suggested to enhance the paper further and align it with publication standards. We appreciate your consideration and valuable input.*

**Reviewer#2, Concern # 2:**

Please give the organization of the paper.

**Author Response:** *Thank you for your positive feedback. We added a paragraph explaining the organization of the manuscript at the end of the Introduction section.*

*In manuscript:*

The organization of this paper is as follows. After the Introduction we give a comprehensive Literature Review. The Methodology section outlines the approach used to apply WOA for feature selection, including data preparation and the experimental setup. Next, we discuss the Experimental Results, showcasing the performance of WOA and other feature selection techniques. The Conclusion summarizes the findings of the study. Finally, we discuss Limitations and Future Studies.

**Reviewer#2, Concern # 3:**

Line 87 onwards: Please mention the paper author instead of just mentioning the reference numbers.

**Author Response:** *Thank you for your valuable feedback. We considered your suggestion in the citation and edited it to refer more directly to the content of the study with the author's name(s) as you suggested.*

**Reviewer#2, Concern # 4:**

Line 153: Please mention the reference papers of WOA. Als mention why WOA is suitable for the problem being solved.

**Author Response:** *Thank you for your valuable suggestions on this subject. We added the reference for WOA. We also added an additional paragraph on why we chose the WOA algorithm for feature selection among other optimization algorithms in this churn study.*

*In manuscript:*

In handling intricate optimization problems, Whale Optimization Algorithm (WOA) presents particular advantages compared to other optimization algorithms. First, WOA is more memory-efficient, as it only stores the best global solution at each iteration, reducing computational overhead [74]. Additionally, WOA exhibits better exploratory capabilities, enabling it to avoid local optima more effectively, which is crucial for problems with many local solutions [75]. Its adaptive mechanisms allow for accelerated exploitation based on iteration progress, leading to more accurate and refined solutions. Unlike many traditional algorithms, which rely on mechanisms such as crossover or mutation, WOA employs mathematical equations to update solutions, resulting in a more stable and efficient optimization process [74], [76]. Furthermore, studies have shown that WOA offers faster convergence and higher solution quality than other optimization algorithms [75].

**Reviewer#2, Concern # 5:**

Table 3: Please write a paragraph explaining in detail. The table is not sufficient for readers who are not familiar with metaheuristics

**Author Response:** *Thank you for your positive feedback. We updated Table 3 with additional information about the formulas used in WOA*

*In manuscript:*

The mathematical model of the three main steps of WOA is presented in Table 3, and the illustration of Bubble Net Attacking is shown in Figure 3.

- Encircling the prey: WOA assumes that the current best candidate solution represents the target prey or is near the optimal solution. Once the best search agent is identified, the other agents update their positions toward it. In this process, t represents the current iteration, A and C are coefficient vectors, Xgb denotes the position of the best solution found so far, and X is the position vector. The position of Xgb is updated in each iteration if a better solution is found. For the calculation of A, a1 decreases linearly from 2 to 0 over the course of the iterations, and r is a random vector in the range [0,1]. By adjusting the values of the A and C vectors, agents can explore different areas around the best solution. Search agents update their positions near the current best solution, simulating the encircling behavior of whales [64].
- Bubble-Net Attacking: To model bubble net behaviour, WOA uses a 50% probability to randomly choose between the shrinking encircling mechanism and the spiral model when updating the whale's position. The shrinking encircling mechanism is achieved by gradually decreasing the value of a. In the spiral updating position, the algorithm first calculates the distance between the current position of the search agent and the best solution. A spiral equation is then applied to mimic the helix-shaped movement of humpback whales. Here, E represents the distance of the i-th whale to the best solution found so far, b is a constant that defines the shape of the logarithmic spiral, l is a random number in the range [−1,1] [64].
- Exploration Phase: In the exploration phase, the variation of A is utilized to search for prey. Humpback whales search randomly based on the positions of other whales. To simulate this, random values for A are set to be greater than 1 or less than -1, enabling whales to move farther away from a reference whale. In contrast to the exploitation phase, the position of a search agent is updated based on a randomly chosen whale from the population rather than the best solution found so far. Xr is a random position vector representing a whale chosen from the current population [64].
